# Socioeconomic and ethnic differences in the relation between dietary costs and dietary quality: the HELIUS study

**DOI:** 10.1186/s12937-019-0445-3

**Published:** 2019-03-28

**Authors:** Joreintje D. Mackenbach, S. Coosje Dijkstra, Joline W. J. Beulens, Jacob C. Seidell, Marieke B. Snijder, Karien Stronks, Pablo Monsivais, Mary Nicolaou

**Affiliations:** 1 Department of Epidemiology and Biostatistics, Amsterdam Public Health research institute, Amsterdam UMC, location VUmc, de Boelelaan 1117, Amsterdam, the Netherlands; 2 Department of Health Sciences, Amsterdam Public Health research institute, Vrije Universiteit, Amsterdam, the Netherlands; 3 Julius Center for Health Sciences and Primary Care, University Medical Center Utrecht, Utrecht, The Netherlands; 4 Department of Public Health, Amsterdam Public Health research institute, Amsterdam UMC, location AMC, Meibergdreef 9, Amsterdam, the Netherlands; 5 Department of Nutrition and Exercise Physiology, Elson S. Floyd College of Medicine, Washington State University, Spokane, USA

**Keywords:** Diet, Dietary costs, DASH, Mediterranean diet, Dutch healthy eating index, Food cost, Ethnicity, Socioeconomic position, HELIUS study

## Abstract

**Background:**

Healthier dietary patterns are generally more costly than less healthy patterns, but dietary costs may be more important for dietary quality in lower educated and ethnic minority groups. The aim of this study was to investigate the association between dietary costs and dietary quality and interactions with ethnicity and socioeconomic position (SEP).

**Methods:**

We used cross-sectional data from 4717 Dutch, Surinamese, Turkish and Moroccan origin participants of the multi-ethnic HELIUS study (the Netherlands), who completed an ethnic-specific food frequency questionnaire (FFQ). The primary outcome measure was dietary quality according to adherence to the Dutch Healthy Diet index 2015 (DHD15-index, range 0–130). Individual dietary costs (the monetary value attached to consumed diets in Euros) were estimated by merging a food price variable with the FFQ nutrient composition database. Regression analyses were used to examine main and interaction effects. Analyses were adjusted for age, sex, smoking, energy intake, physical activity, ethnicity and educational level.

**Results:**

Having higher dietary costs was associated with higher dietary quality. Analyses stratified by educational level showed that associations were stronger in higher educated (B_tertile3_ = 8.06, 95%CI = 5.63; 10.48) than in lower educated participants (B_tertile3_ = 5.09, 95%CI = 2.74; 7.44). Stratification by ethnic origin showed strongest associations in Turkish participants (B_tertile2_ = 9.31, 95%CI = 5.96; 12.65) and weakest associations in Moroccan participants (B_tertile3_ = 4.29, 95%CI = 0.58; 8.01). Regardless of their level of education, Turkish and Moroccan individuals consumed higher quality diets at the lowest cost than Dutch participants.

**Conclusions:**

The importance of dietary costs for dietary quality differs between socioeconomic and ethnic subgroups. Increasing individual food budgets or decreasing food prices may be effective for the promotion of healthy diets, but differential effects across socioeconomic and ethnic subgroups may be expected.

**Electronic supplementary material:**

The online version of this article (10.1186/s12937-019-0445-3) contains supplementary material, which is available to authorized users.

## Background

The promotion of healthy diets is integral to population- level strategies aiming to reduce the burden of chronic disease [1]. However, an important barrier for the consumption of a healthy diet is the higher price of healthy food [2, 3]. Several studies have shown that the nutritional quality of diets is positively associated with their monetary costs [4–6]. It is suggested that this is due to the lower cost of energy-dense foods compared to less energy-dense foods, as sugar and fat are relatively cheap sources of energy [7–9].

Recent evidence suggests that food pricing strategies such as taxes and subsidies may be effective in promoting healthier diets [10, 11]. Yet, their effectiveness may differ between subgroups depending on the relative importance of dietary costs for dietary quality.

Dietary costs as a constraint on dietary quality could be especially crucial for lower socio-economic position (SEP) populations, who tend to prioritize low-cost in food choices and may lack other resources that motivate healthy eating [12, 13]. Two studies in the UK demonstrated that the association between dietary costs and dietary intake was stronger for less-educated and lower income groups [14, 15]. This suggests that, in low SEP subgroups, having high dietary costs may be relatively important for dietary quality, compared to high SEP subgroups that benefit from additional material and psychosocial resources. However, a study conducted in the Netherlands did not provide evidence for an interaction between income and dietary costs in relation to energy density or fruit and vegetable intake [16], suggesting that this association may be country-specific.

The importance of dietary costs for dietary quality may also differ between ethnic subgroups. However, the interaction between dietary costs and ethnicity may be of a different nature than with SEP, despite the fact that ethnic minority populations often have a low SEP. Previous studies have shown that Mexican-American and Hispanic adults were able to achieve higher dietary quality with lower dietary costs than non-Hispanic black and white Americans, independent of SEP [6, 17]. It could be that some ethnic groups may be able to consume a healthier diet at a lower cost due to cultural knowledge and food-related skills that lessens the importance of access to affordable food. Whether this pattern holds in ethnic subgroups outside the US is yet to be determined: it may be that the desire to maintain traditional, culturally-specific food habits leads to higher dietary costs due to greater expense of ingredients that are central to the traditional diet but scarcer in the country of settlement.

In summary, evidence for differences in the relative importance of dietary costs for dietary quality in different socioeconomic and ethnic subgroups is rather limited and mainly based on studies conducted in the United Kingdom and the United States. Insight into the relative importance of dietary costs for dietary quality in different subgroups would provide insight into the potential differential effectiveness of pricing strategies to promote a healthy diet. In the present study, we used data from the Dutch HELIUS study to investigate: 1) the overall association between dietary costs and dietary quality and 2) interactions with ethnicity and SEP. In addition, we investigated: 3) the three-way interaction between dietary costs, SEP and ethnicity in relation to dietary quality to examine which subgroups are able to achieve a relatively high dietary quality at low dietary costs.

## Methods

### Study design

The aims and design of the HELIUS study have been described in detail elsewhere [18, 19]. In brief, HELIUS is a large-scale prospective cohort study on health and health care utilization among different ethnic groups living in Amsterdam, the Netherlands. Baseline data collection took place in 2011–2015 among nearly 25.000 participants (aged 18–70 years) of Dutch, Surinamese, Turkish, Moroccan and Ghanaian origin people. Data were collected by questionnaires and a physical examination. The study protocols were approved by the Ethical Review Board of the Amsterdam Medical Center, and all participants provided written informed consent.

The current study is a sub-sample of Dutch, Surinamese, Turkish and Moroccan participants who agreed to also take part in the HELIUS-dietary patterns sub-study and included 5358 participants [20]. Participants with other/unknown ethnic origin (*n* = 11) or ‘other’ Surinamese origin (*n* = 148) (see definition ethnicity below), individuals with extreme energy values (< 500 or > 3500 for women and < 800 or > 4000 for men [21]; *n* = 318) and individuals with missing values on covariates (*n* = 164) were excluded, resulting in an analytical sample of *n* = 4717.

### Outcome variables - dietary quality

Four ethnic-specific Food Frequency Questionnaires (FFQs) specially developed for the HELIUS study were used to collect usual dietary intake data for Dutch, Surinamese, Turkish and Moroccan ethnic groups [22]. The FFQs were based on an existing, validated 183-item semi quantitative Dutch FFQ, which has shown an acceptable to good ability to rank subjects on most nutrients and foods [23]. Each FFQ included questions on the frequency and portion size of approximately 200 food items eaten during the past month.

Dietary quality was evaluated by using the Dutch Healthy Diet index 2015 (DHD15, [24–26]), which reflects adherence to the Dutch dietary guidelines 2015. The index consists of 15 components representing the food-based Dutch dietary guidelines of 2015 and includes vegetables, fruits, wholegrain products, legumes, nuts, dairy, fish, tea, fats and oils, coffee, red meat, processed meat, sweetened beverages and fruit juices, alcohol and salt. Per component the score ranges from 0 to 10, resulting in a total score between 0 (no adherence) to 150 (complete adherence). Because our FFQs did not allow for the assessment of intake of coffee and tea separately, or for the intake of salt, these two components were left out of the calculations, resulting in potential scores between 0 and 130. The top quintile (DHD15-index> 96) was used to indicate ‘high’ dietary quality according to the DHD15-index.

In sensitivity analyses, we repeated analyses with the DHD15-index excluding the alcohol component, to ascertain that any observed associations would not be driven by the large ethnic [27, 28] and socioeconomic [29] differences in alcohol consumption habits and costs associated with alcohol consumption. In addition, we used two additional, frequently-used dietary quality indicators, namely dietary accordance with the DASH diet [30–32], and the Mediterranean Diet Score (MDS) [33–35]. The construction of these indicators is described in more detail in Additional file 1. These primary endpoints were pre-defined and did not change during the course of the post-hoc analyses.

### Explanatory variable - dietary costs

We used established techniques to derive the monetary costs of diets [36, 37], as described in detail in Additional file 2. Individual dietary costs (the monetary value of consumed diets, in Euros) were estimated by merging a food price variable with the FFQ nutrient composition database [36]. In total, the four HELIUS FFQs consisted of 1247 unique foods. Each individual food product underlying the FFQ-items was translated to a specific food item in purchasable form. For example, ‘apple without peel’ was translated into ‘apple’. In addition, for pragmatic reasons, food items that essentially represented the same products were combined (e.g., different types of concentrated fruit cordials). This resulted in a list of 902 food products for which we collected retail price data on in the summer of 2017 (from two key supermarket chains (one discount and one regular supermarket) and several ethnic supermarkets and local shops such as bakeries, butchers, fish mongers, etc.) in Amsterdam. For each product, the lowest, non-promotion price was selected. For packaged foods, the medium package size was selected. Prices were adjusted for preparation and waste [37] to yield an adjusted food price for each 100 g edible portion [38]. Combining this new food price variable with the HELIUS food and nutrient database allowed the estimation of the monetary value of each participant’s diet. The variable obtained for each respondent was dietary costs per day (€/day – crude diet cost) and dietary cost per 2000 kcal. The dietary cost per 2000 kcal reflects a daily energy ratio for many adults and has been used widely in the literature (e.g. [6, 39, 40]).

### Moderating variables – ethnicity and SEP

Ethnic origin was defined according to the country of birth of the participant as well as that of his/her parents [41]. Specifically, a participant was considered as of non-Dutch ethnic origin if she/he fulfilled either of the following criteria: 1) she/he was born abroad and has at least one of her/his parents born abroad (first generation); or 2) she/he was born in the Netherlands but both his/her parents were born abroad (second generation). After data collection, participants of Surinamese ethnic origin were further classified according to self-reported ethnic origin (obtained by questionnaire) into ‘African Surinamese’, ‘South-Asian Surinamese’ or ‘other’. For the Dutch sample, we invited people who were born in the Netherlands and whose parents were born in the Netherlands.

We used educational level as an indicator of SEP. Educational level was based on the highest qualification obtained either in the Netherlands or in the country of origin. This variable was classified into low education (never been to school or elementary schooling, lower vocational schooling or lower secondary schooling only), medium education (intermediate vocational schooling or intermediate/higher secondary education schooling) and high education (higher vocational schooling or university).

### Covariates

Covariates were determined a priori and included age, sex, energy intake, physical activity and smoking. Energy intake was derived from the FFQs and defined as kcal per day. Self-reported habitual physical activity was measured using the Short Questionnaire to Assess Health (SQUASH) [42] and converted into minutes per week spent in light and moderate/high intensity activities based on age-specific Metabolic Equivalent Tasks (METs) derived from Ainsworth’s compendium of physical activity [43]. Smoking status was defined as current smoker, never smoker or former smoker.

### Statistical analysis

Participant characteristics were described using percentages for categorical variables and means with standard deviations or medians with interquartile ranges for continuous variables. In addition, we conducted a General Linear Model Univariate procedure with estimated marginal means adjusted for age, sex, education, smoking, physical activity and energy intake to derive mean DHD15-index scores for each ethnic group.

As the DHD15 score was normally distributed, we assessed the association between daily dietary costs and the DHD15 score (study aim 1) using linear regression analysis, only adjusted for energy intake (model 1) and additionally adjusted for age, sex, smoking status, education, ethnicity and physical activity (model 2). Analysis with a continuous measure of daily dietary costs and its quadratic term suggested that the association with the dietary quality indicators was not linear but concave, so we analyzed the daily dietary costs as a categorical predictor variable using tertiles. Logistic regression analyses were used to examine the association between tertiles of daily dietary costs and having a high dietary quality.

To test whether the association between dietary costs and the DHD15 score differed between ethnic and socioeconomic groups (study aim 2), we examined the interaction between ethnicity and dietary costs, and education and dietary costs by adding a cross-product of these variables to the regression models. Following significant interaction, analyses were stratified by education or ethnicity.

To investigate which subgroups are able to achieve a relatively high dietary quality at low dietary costs (study aim 3), we examined the three-way interaction between dietary costs, education and ethnicity. We conducted a General Linear Model Univariate procedure with estimated marginal means to graphically display differences in dietary quality across subgroups.

All analyses were conducted using SPSS version 23.0, and statistical significance was defined as *p* < .05.

## Results

The population characteristics are presented in Table 1. Briefly, the average age was 46.5 years and 40.3% were men. Most (61.9%) of the participants of Dutch origin had a high level of education, while this percentage was much lower for the participants of South Asian Surinamese (25.8%), African Surinamese (28.7%), Moroccan (21.6%) and Turkish (19.5%) origin. Participants of Turkish origin had the highest dietary costs (daily and per 2000 kcal) and participants of Moroccan origin had the lowest dietary costs. Crude and adjusted mean dietary quality was highest in the participants of Moroccan origin and lowest in those of Dutch origin.

**Table 1:** Characteristics of the study participants, by ethnic origin (*n* = 4717)

	Ethnic origin					All (*n* = 4717)
	Dutch (*n* = 1429)	South-Asian Surinamese (*n* = 1003)	African Surinamese (*n* = 980)	Moroccan (*n* = 717)	Turkish (*n* = 588)	
Age (years)	48.2 (13.5)	47.6 (12.2)	49.7 (11.2)	41.1 (12.0)	41.7 (11.0)	46.5 (12.6)
Men (n (%) of participants)	635 (44.2%)	408 (40.7%)	314 (32.0%)	270 (37.7%)	277 (47.1%)	1904 (40.4%)
N (%) of participants with lowest educational level	238 (16.7%)	463 (46.2%)	362 (36.9%)	333 (46.4%)	286 (48.6%)	1682 (35.7%)
N (%) of participants with medium education level	306 (21.4%)	281 (28.0%)	337 (34.4%)	244 (34.0%)	175 (29.8%)	1343 (28.5%)
N (%) of participants with highest education level	885 (61.9%)	259 (25.8%)	281 (28.7%)	140 (19.5%)	127 (21.6%)	1692 (35.9%)
Physical activity (total MET-minutes/week, median (IQR))	7512 (5510; 9818)	6372 (4124; 9375)	6960 (4440; 10,883)	5550 (3210; 8415)	5430 (2527; 8666)	6654 (4230–9502)
Current smoker (n (%) of participants)	325 (22.7%)	235 (23.4%)	224 (22.9%)	76 (10.6%)	167 (28.4%)	1027 (21.8%)
Daily dietary costs (€/day)	5.64 (1.48)	5.00 (1.78)	5.16 (1.88)	4.78 (1.73)	5.91 (2.05)	5.31 (1.79)
Energy adjusted dietary costs (€/2000 kcal)	5.31 (1.15)	5.19 (1.25)	5.24 (1.57)	4.79 (1.34)	5.64 (1.53)	5.23 (1.37)
DHD15-index, crude mean	78.2 (16.4)	83.4 (16.9)	79.3 (16.1)	86.5 (16.1)	84.4 (15.7)	81.5 (16.6)
High diet quality (% top quintile)	12.9%	24.7%	16.1%	31.7%	22.1%	20.1%
DHD15-index, adjusted mean (95% CI)^a^	76.8 (75.9; 77.6)	83.8 (82.8; 84.7)	77.8 (76.9; 78.8)	88.1 (86.9; 89.2)	87.5 (86.3; 88.8)	–

Data are mean (standard deviation) unless otherwise stated

*IQR* Inter quartile range, *DHD* Dutch Healthy Diet, *CI* Confidence Interval, *MET* metabolic equivalent of task

^a^Means and 95% CIs were derived from a General Linear Model Univariate procedure with estimated marginal means and are adjusted for age, sex, education, energy intake, smoking, physical activity

Table 2 displays the association between dietary costs and dietary quality. Individuals with medium or high dietary costs (tertile 2 and 3) had a 6.7 point higher DHD15-index or a 2.6 times higher odds of having a high dietary quality compared to individuals with low dietary costs. In analyses excluding alcohol consumption from the DHD15-index (Additional file 3**:** Table S1), a slight gradient across tertiles was observed, and coefficients were a bit larger. Analyses using the MDS or DASH score as dietary quality indicator (Additional file 3**:** Table S2) showed similar results, albeit with much larger coefficients and a stronger gradient in analyses with the MDS.

**Table 2:** Association between daily dietary costs in tertiles and (high) dietary quality (*n* = 4717)

Dietary quality (DHD15-index – continuous score)		Model 1 ^a^		Model 2 ^b^	
		β	95% CI	β	95% CI
Dietary costs	T1 (1,14-4,56€)	ref		ref	
	T2 (4,57-5,58€)	6.54^**^	5.29; 7.80	6.69^**^	5.52; 7.86
	T3 (5,59-17,15€)	7.13^**^	5.59; 8.67	6.70^**^	5.25; 8.15
High dietary quality (DHD15-index – dichotomous)		Model 1 ^a^		Model 2 ^b^	
		OR	95% CI	OR	95% CI
Dietary costs	T1 (1,14-4,56€)	ref		ref	
	T2 (4,57-5,58€)	2.16^**^	1.77; 2.63	2.60^**^	2.10; 3.22
	T3 (5,59-17,15€)	2.24^**^	1.76; 2.86	2.64^**^	2.02; 3.44

High dietary quality was defined as the top quintile

*DHD15-index* Dutch Healthy Diet index 2015, *T* Tertile, *Ref* reference group, *CI* Confidence Interval, *OR* Odds ratio

^*^P < 0.05; ^**^P < 0.001

^a^ Model 1 is only adjusted for dietary energy | ^b^ Model 2 additionally adjusts for age, sex, education, ethnicity, smoking and physical activity

As all cross-product terms of the interaction between ethnicity and dietary costs and education and dietary costs in the association with DHD15 were statistically significant, analyses were stratified by education and ethnicity. The associations between dietary costs and the DHD15-index were relatively comparable across education groups (Table 3), but slightly stronger in the high educated group in particular. For example, in participants with low education, the top tertile of dietary costs was associated with a 5-point higher DHD15-index than for those in the lowest tertile of costs, while among participants with high education, there was an 8-point difference in DHD15-index between those in the highest and lowest tertiles of dietary costs. In the high educated individuals, there was a clear gradient (higher dietary quality across higher tertiles of dietary costs), while this was not observed in medium educated individuals, and a reversed gradient in the lowest educated individuals. Excluding the alcohol component from the DHD15-index resulted in similar but somewhat stronger associations. Analyses with MDS and DASH as outcomes (Additional file 3**:** Table S4) showed clear gradients across all education groups, and stronger associations in the medium educated group.

**Table 3:** Association between daily dietary costs in tertiles and (high) dietary quality, by level of education (*n* = 4717)

Dietary quality (DHD15-index – continuous score)		Lowest educated (*n* = 1682)		Medium educated (*n* = 1343)		Highest educated (*n* = 1692)	
		β	95% CI	β	95% CI	β	95% CI
Dietary costs	T1 (1,14-4,56€)	ref		ref		ref	
	T2 (4,57-5,58€)	6.55^**^	4.63; 8.47	6.91^**^	1.35; 5.26	6.59^**^	4.63; 8.56
	T3 (5,59-17,15€)	5.09^**^	2.74; 7.44	6.96^**^	3.49; 8.06	8.06^**^	5.63; 10.48
High dietary quality (DHD15-index – dichotomous)		OR	95%CI	OR	95%CI	OR	95%CI
Dietary costs	T1 (1,14-4,56€)	ref		ref		ref	
	T2 (4,57-5,58€)	2.79^**^	2.00; 3.94	2.30^**^	1.54; 3.43	2.88^**^	1.96; 4.25
	T3 (5,59-17,15€)	2.37^**^	1.54; 3.65	2.10^*^	1.27; 3.48	3.72^**^	2.35; 5.90

High dietary quality was defined as the top quintile

All models adjust for age, sex, ethnicity, smoking, energy intake and physical activity

*DHD15-index* Dutch Healthy Diet index 2015, *T* Tertile, *Ref* reference group, *CI* Confidence Interval

^*^P < 0.05; ^**^P < 0.001

Table 4 shows that the associations between dietary costs and the DHD15-index were similar in direction across ethnic groups, but strongest in Turkish participants. Only in Dutch origin participants, a clear gradient in dietary quality across tertiles of dietary costs was observed. Excluding the alcohol component from the DHD15-index resulted in stronger associations and stronger gradients across dietary costs (Additional file 3**:** Table S5). Analyses with MDS and DASH as outcomes (Additional file 3: Table S6) showed clear gradients across all ethnic groups, and strongest associations in the South-Asian Surinamese group.

**Table 4:** Association between daily dietary costs in tertiles and dietary quality, by ethnicity (*n* = 4717)

Dietary quality (DHD15-index – continuous score)		Dutch (*n* = 1429)		South-Asian Surinamese (*n* = 1003)		African Surinamese (*n* = 980)		Moroccan (*n* = 717)		Turkish (*n* = 588)	
		β	95% CI	Β	95% CI	β	95% CI	β	95% CI	β	95% CI
Dietary costs	T1 (1,14-4,56€)	ref		ref		ref		ref		ref	
	T2 (4,57-5,58€)	4.63^**^	2.39; 6.88	6.29^**^	3.80; 8.79	7.67^**^	5.20; 10.15	5.87^**^	2.96; 8.78	9.31^**^	5.96; 12.65
	T3 (5,59-17,15€)	6.00^**^	3.22; 8,78	6.89^**^	3.62; 10.16	7.84^**^	4.81; 10.87	4.29^*^	0.58; 8.01	7.50^**^	3.73; 11.26
High dietary quality (DHD15-index – dichotomous)		OR	95% CI	OR	95% CI	OR	95% CI	OR	95% CI	OR	95% CI
Dietary costs	T1 (1,14-4,56€)	ref		ref		ref		ref		ref	
	T2 (4,57-5,58€)	1.86^*^	1.11; 3.11	2.42^**^	1.59; 3.69	4.07^**^	2.45; 6.73	2.13^*^	1.38; 3.31	4.84^**^	2.35; 9.99
	T3 (5,59-17,15€)	2.77^*^	1.51; 5.06	2.79^**^	1.61; 4.83	3.28^**^	1.78; 6.03	1.11	0.62; 1.99	5.67^**^	2.56; 12.56

High dietary quality was defined as the top quintile

All models adjust for age, sex, education, smoking, energy intake and physical activity

*DHD15-index* Dutch Healthy Diet index 2015, *T* Tertile, *Ref* reference group, *CI* Confidence Interval

^*^P < 0.05; ^**^P < 0.001

**Fig. 1:**
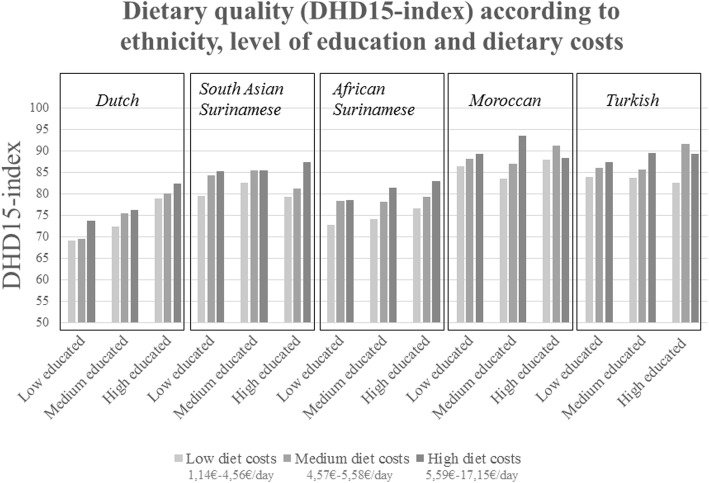
Dietary quality (DHD15-index) according to ethnicity, level of education, and dietary costs

A three-way interaction was not statistically significant, but interactions between daily dietary costs and ethnicity, and between ethnicity and level of education, were. Figure 1 displays the estimated marginal DHD15-index means according to ethnicity, level of education, and daily dietary costs. At the lowest dietary costs, Moroccan and Turkish origin participants consumed the healthiest diets -regardless their level of education, together with medium educated South-Asian Surinamese origin participants. For instance, low educated Moroccan participants with low dietary costs had an average DHD15-index of 86.4 and low educated Dutch participants with low dietary costs had an average DHD15-index of 69.2. In addition, Fig. 1 shows that, in Dutch and African Surinamese participants, both higher level of education and higher level of dietary costs were associated with higher dietary quality. This is in contrast to South-Asian Surinamese participants, for whom higher dietary costs, but not higher educational level, was associated with higher dietary quality. In Moroccan and Turkish participants, higher education was not clearly associated with higher level of dietary quality, and higher level of dietary costs was only associated with higher level of dietary quality among low and medium educated participants.

## Discussion

In this multi-ethnic cohort in the Netherlands, we examined the overall association between dietary costs and dietary quality and their interactions with ethnicity and SEP, and studied which subgroups are able to achieve a relatively high dietary quality at low dietary costs.

First, in accordance with previous studies [4–6], we observed that higher dietary costs were consistently associated with higher dietary quality, regardless the dietary indicator that we used – confirming the robustness of these findings. Individuals in the highest tertile of dietary costs had 2.6 times the odds of having high dietary quality as defined by the top quintile of the DHD15-index. A previous study has shown that participants in the top quintile of the DHD15-index had a 14% lower mortality risk, 26% lower risk of stroke, 28% lower risk of COPD and 29% lower risk of colorectal cancer, than participants in the lowest quintile [44]. Consequently, our results confirm the notion that besides nutrition knowledge, material resources, accessibility and availability of foods, the cost or price of foods may be an important component of interventions and policies aiming to improve population diets and prevent diet-related chronic diseases [45, 46].

Second, the importance of dietary costs for dietary quality differed between socioeconomic and ethnic subgroups. We found that the association between dietary costs and dietary quality was somewhat stronger in medium and high educated groups -with some differences between dietary quality indicators, which is in contrast to the findings of the previous UK and Dutch studies [14–16]. It could be speculated that higher educated groups use their money to improve dietary quality because of their nutrition knowledge. Also, depending on the dietary quality indicator used, South- Asian Surinamese, African Surinamese and Turkish participants showed the strongest association between dietary costs and dietary quality, irrespective of their level of education. However, there is a strong interplay between educational level and ethnicity in this cohort, and Fig. 1 reveals that for the Dutch origin participants, the association between dietary costs and dietary quality was stronger in those with lower education. While for African Surinamese and Moroccan participants, the association was stronger in those with medium education, and for South-Asian Surinamese and Turkish participants in those with higher education. A proportion of the ethnic minority groups obtained their education in their country of origin and it may be speculated that obtaining education elsewhere does not result in the same benefits as obtaining education in the new country. I.e., the material and psychosocial resources associated with high education in Dutch participants may diminish the relative importance of dietary costs, while the resources associated with high education in South-Asian Surinamese and Turkish participants result in a higher relative importance of dietary costs.

Third, we examined which subgroups are able to achieve a relatively high dietary quality at low dietary costs. Studies in the US demonstrated that Mexican-American and Hispanic adults were able to achieve higher dietary quality with lower dietary costs than non-Hispanic black and white Americans [6, 17]. Similarly, we observed that at the lowest dietary costs, Moroccan, Turkish and South- Asian Surinamese origin participants consumed healthier diets than Dutch and African Surinamese origin participants. Dutch individuals had the lowest dietary quality overall; e.g., high educated Dutch individuals still had a lower dietary quality than low educated Moroccan individuals. Future studies should examine what dietary components (e.g., lower consumption of processed foods) and what alternative resources (e.g., cultural knowledge, cooking skills) explain the higher dietary quality of Moroccan individuals at a low cost, independent of level of education, to better inform strategies to improve population diets.

The results of this study should be viewed in the light of some limitations. FFQs have known biases, and the FFQs used may both have under- and overestimated absolute intake of various foods [23]. A major strength of this study, however, was the use of ethnic-specific FFQs which accounted for the consumption of ethnic-specific foods otherwise not adequately captured in regular FFQs. In addition, the estimation of dietary costs based on the method of food retail prices to dietary intake is not without ambiguity [47], since dietary quality may both be a consequence as well as a predictor of dietary costs [48]. However, dietary costs (as derived from reported dietary intakes and a fixed database of food prices) are modestly but positively correlated with actual food spending [36, 49] and therefore suitable for our purpose of ranking individuals into tertiles of dietary cost. Also, comparing the average daily food costs (which were based on the lowest available prices) in this study (€5.31) to national data on average daily food spending (6,50€, based on mean prices [50]) suggests that our measure of dietary costs is a relatively good indicator of food spending. Moreover, our conservative approach of only using lowest available prices is more likely to have led to an underestimation than to an overestimation of the association between dietary costs and dietary quality.

## Conclusion

In conclusion, higher dietary costs are consistently associated with higher dietary quality. The results of this study suggest that if interventions or policies targeting food prices were to be implemented for the promotion of healthy diets, individuals across a range of educational and ethnic backgrounds would benefit from these measures. However, as the strength of the association differed between socioeconomic and ethnic subgroups, some subgroups, such as lower educated Dutch individuals or high educated South-Asian Surinamese individuals in this study, may be expected to benefit more. Given that some subgroups were better able to achieve a high dietary quality at a low dietary cost, insight into the interaction between a range of resources -including nutrition knowledge, material resources, accessibility and availability of food retailers, the cost of foods and cultural skills and knowledge- to dietary quality is needed. This may be especially important for groups with a relatively low diet quality despite high dietary costs.

## Additional files

Additional file 1: Derivation of two additional dietary quality indicators. (DOCX 20 kb)

Additional file 2: Methodology used to derive the lowest available food prices for participants of the HELIUS study. (DOCX 16 kb)

Additional file 3: **Table S1.** Association between daily dietary costs in tertiles and (high) dietary quality excluding the alcohol component (*n* = 4717). **Table S2.** Association between daily dietary costs and dietary quality according to the MDS and DASH scores (*n* = 4717). **Table S3.** Association between daily dietary costs in tertiles and (high) dietary quality excluding the alcohol component, by level of education (*n* = 4717). **Table S4.** Association between daily dietary costs and dietary quality according to the MDS and DASH scores by level of education (*n* = 4717). **Table S5.** Association between daily dietary costs in tertiles and dietary quality excluding the alcohol component, by ethnicity (*n* = 4717). **Table S6.** Association between dietary costs and dietary quality according to the MDS and DASH scores by ethnicity (*n* = 4717). (DOCX 33 kb)

## Abbreviations

DASH: Dietary Approaches to Stop Hypertension
DHD15-index: Dutch Healthy Diet index 15
FFQ: Food frequency questionnaire
HELIUS: Healthy Life in an Urban Setting
MDS: Mediterranean Diet Score
SEP: Socioeconomic position
