# Supplementary material for: Socioeconomic and ethnic differences in the relation between dietary costs and dietary quality: the HELIUS study
Source: Nutr J. 2019 Mar 28;18:21. doi: 10.1186/s12937-019-0445-3 (PMC6440156; doi:10.1186/s12937-019-0445-3)
Supplement: Supplementary file 1 — Derivation of two additional dietary quality indicators. (DOCX 20 kb) [file 12937_2019_445_MOESM1_ESM.docx]

**Additional File 1. Derivation of two additional dietary quality indicators.**

In sensitivity analyses, we used two additional frequently-used dietary quality indicators. Firstly, dietary accordance with the DASH diet(1–3), adapted from that of Fung et al(4,5). This index consists of eight dietary components (grains/ grain products; vegetables; fruits; low fat/ fat-free dairy; red and processed meat; nuts/ seeds/ dry beans; dietary sodium; and foods high in added sugar)(1). As our FFQ’s did not allow for accurate data collection on sodium intake, this component was excluded in the current study. Energy-adjusted residuals(6) of the seven remaining components were divided into quintiles. By summing individual quintile scores the overall DASH scores ranged between 7 (least healthy) and 35 (most healthy). DASH accordance was defined as a DASH score in the top quintile (>24 in this cohort)(5,7). In addition, we used the Mediterranean Diet Score (MDS) indicating compliance to the Mediterranean diet(8–10). The MDS as applied in this study followed the recommendations of Panagiotakos et al.(11). For the consumption of items presumed to be close to the Mediterranean dietary patterns (non-refined cereals, fruits, vegetables, legumes, olive oil, fish and potatoes) scores of zero to five for never, rare, frequent, very frequent, weekly and daily consumption were assigned, while for the consumed of foods presumed to be farther away from this pattern (red meat and products, poultry and full fat dairy products) scores were assigned on a reverse scale. As a result, after summing the individual component scores, the overall MDS ranged from zero (lowest compliance) to 80 (highest compliance).

**References**

1. Karanja N, Erlinger TP, Pao-Hwa L, Miller ER, Bray GA. The DASH diet for high blood pressure: from clinical trial to dinner table. Cleve Clin J Med. 2004;71:745–53.

2. Chiu S, Bergeron N, Williams PT, Bray GA, Sutherland B, Krauss RM. Comparison of the DASH (Dietary Approaches to Stop Hypertension) diet and a higher-fat DASH diet on blood pressure and lipids and lipoproteins: a randomized controlled trial. Am J Clin Nutr. 2016;103:341–7.

3. Salehi-Abarqouei A, Maghsoudi Z, Shirani F, Azadbakht L. Effects of Dietary Approaches to Stop Hypertension (DASH)-style diet on fatal or nonfatal cardiovascular disease-incidence: a systematic review and meta-analysis on observational prospective studies. Nutrition. 2013;29:611–8.

4. Monsivais P, Scarborough P, Lloyd T, Mizdrak A, Luben R, Mulligan AA, Wareham NJ. Greater accordance with the Dietary Approaches to Stop Hypertension dietary pattern is associated with lower diet-related greenhouse gas production but higher dietary costs in the United Kingdom. Am J Clin Nutr. 2015;102:138–45.

5. Fung TT, Chiuve SE, McCullough ML, Rexrode KM, Logroscino G, Hu FB. Adherence to a DASH-style diet and risk of coronary heart disease and stroke in women. Arch Intern Med. 2008;168:713–20.

6. Willett WC, Howe R. Adjustment for total energy intake in epidemiologic studies. Cancer. 1997;65:1220S–1228S.

7. Sherzai A, Heim LT, Boothby C, Sherzai a D. Stroke, food groups, and dietary patterns: a systematic review. Nutr Rev. 2012;70:423–35.

8. Trichopoulou A, Orfanos P, Norat T, et al. Modified Mediterranean diet and survival: EPIC-elderly prospective cohort study. Bmj. 2005;330:991.

9. Bach A, Serra-Majem L, Carrasco JL, Roman B, Ngo J, Bertomeu I, Obrador B. The use of indexes evaluating the adherence to the Mediterranean diet in epidemiological studies: a review. Public Health Nutr. 2006;9:132–46.

10. Sofi F, Cesari F, Abbate R, Gensini GF, Casini A. Adherence to Mediterranean diet and health status: meta-analysis. Bmj. 2008;337:a1344.

11. Panagiotakos DB, Pitsavos C, Arvaniti F, Stefanadis C. Adherence to the Mediterranean food pattern predicts the prevalence of hypertension, hypercholesterolemia, diabetes and obesity, among healthy adults; the accuracy of the MedDietScore. Prev Med (Baltim). 2007;335–340.
