# Supplementary material for: Socioeconomic and ethnic differences in the relation between dietary costs and dietary quality: the HELIUS study
Source: Nutr J. 2019 Mar 28;18:21. doi: 10.1186/s12937-019-0445-3 (PMC6440156; doi:10.1186/s12937-019-0445-3)
Supplement: Supplementary file 2 — Methodology used to derive the lowest available food prices for participants of the HELIUS study. (DOCX 16 kb) [file 12937_2019_445_MOESM2_ESM.docx]

**Additional File 2. Methodology used to derive the lowest available food prices for participants of the HELIUS study.**

A more detailed description of this methodology is available in the methodology report which can be downloaded from this [website](https://www.amsterdamresearch.org/web/public-health/research-1/health-behaviors-chronic-diseases/2017-1.htm).

Briefly, we identified food retailers that represented a wide range of what is available in Amsterdam, the Netherlands, which included a discount supermarket chain (Lidl), a full-service supermarket chain (Albert Heijn) and local (ethnic) shops such as a Turkish butcher and greengrocer. In total, prices were collected in 20 shops during July and August of 2017 (to prevent variation in food prices due to seasonality) and to be able to obtain the lowest price available in Amsterdam. We used Excel-sheets to collect the food price data. In total, the four HELIUS Food Frequency Questionnaires (FFQs) consisted of 1247 unique foods. Each individual food product underlying the FFQ-items was translated to a specific food item in purchasable form. For example, ‘apple without peel’ was translated into ‘apple’. In addition, for pragmatic reasons, food items that essentially represented the same products were combined (e.g. different types of concentrated cordials). This resulted in a list of 902 food products for which we collected food price data. Foods were sorted according to food group to facilitate speedy data collection. Data were collected on food price (€), unit (in KG or L), name of the product, date at which this price was collected and any remarks with regard to the product. For products in tin or glass jars, we used the net weight of the product as unit. For each shop we used a separate excel-sheet. During the in-store data collection, photos were made of each food item, where possible. Like the photos, data were stored on a tablet. Cheapest products were selected in case there were multiple options. For packaged foods, we selected the median package size. When items were on sale, the sale price was ignored and the everyday price was recorded. In rare cases, a product was not available and we identified a close match that served as a substitute food. For one type of fruit (medlars/mispel), no price could be obtained and there was no close substitute available, so this price was missing. After collecting food price data in the selected shops, data on food prices was combined into a single excel sheet with colours indicating the source of the price information. We used formulas to calculate prices per 100 grams based on the price and the unit of the product to avoid human errors. We used the Dutch ”Maten Gewichten en Codenummers 2003”(1) to calculate the edible portion per food. This report also has information on the weights of average non-packaged foods, such as zucchinis, and information on waste and gain/loss during preparation. The food prices of these 902 purchasable food items were then linked to the 1247 FFQ food products included in the HELIUS FFQs. We used the weighting unique to each of the four FFQs to calculate the price in €/100 gr edible portion for each weighted food. For example, the price for the food ‘muesli’ consists of 45.4% ‘natural muesli’, 40.5% ‘muesli with fruit’, 8.3% ‘muesli with nuts’ and 5.8% ‘muesli with chocolate’. For each FFQ, a nutrient database is constructed based on the Dutch Food Composition Table 2014 (Nevo)(2). This nutrient database is used to calculate the nutrient intake of each individual based on their intake of each food product in the FFQ. The food price variable obtained from the steps described above is added to the nutrient database and the code (syntax) used to calculate the nutrient intake of each individual is expanded to include the variable “weighted food cost”.

**References**

1. Donders-Engelen M, van der Heijden L, Hulshof K. Maten Gewichten en Codenummers 2003. Wageningen UR, Vakgroep Humane Voeding.

2. National Institute for Public Health & the Netherlands Nutrition Centre. Dutch Food Composition Table 2011. The Hague; 2011
