# Supplementary material for: Socioeconomic and ethnic differences in the relation between dietary costs and dietary quality: the HELIUS study
Source: Nutr J. 2019 Mar 28;18:21. doi: 10.1186/s12937-019-0445-3 (PMC6440156; doi:10.1186/s12937-019-0445-3)
Supplement: Supplementary file 3 — Table S1. Association between daily dietary costs in tertiles and (high) dietary quality excluding the alcohol component (n = 4717). Table S2. Association between daily dietary costs and dietary quality according to the MDS and DASH scores (n = 4717). Table S3. Association between daily dietary costs in tertiles and (high) dietary quality excluding the alcohol component, by level of education (n = 4717). Table S4. Association between daily dietary costs and dietary quality according to the MDS and DASH scores by level of education (n = 4717). Table S5. Association between daily dietary costs in tertiles and dietary quality excluding the alcohol component, by ethnicity (n = 4717). Table S6. Association between dietary costs and dietary quality according to the MDS and DASH scores by ethnicity (n = 4717). (DOCX 33 kb) [file 12937_2019_445_MOESM3_ESM.docx]

**Additional File 3. Supplementary tables.**

**Table S1:** Association between daily dietary costs in tertiles and (high) dietary quality excluding the alcohol component (n=4717)

| **Dietary quality (DHD15 index – continuous score)** | | Model 1 ^a^ |  | Model 2 ^b^ |  | |
| --- | --- | --- | --- | --- | --- | --- |
|  |  | β | 95% CI | β | 95% CI | |
| Dietary costs | T1 (1,14-4,56€) | ref |  | ref |  | |
|  | T2 (4,57-5,58€) | 8.88** | 7.70; 10.07 | 7.85** | 6.72; 8.98 | |
|  | T3 (5,59-17,15€) | 10.18** | 8.72; 11.64 | 8.60** | 7.20; 10.01 | |
|  | |  |  |  |  | |
| **High dietary quality (DHD15 index – dichotomous)** | | Model 1 ^a^ |  | Model 2 ^b^ |  | |
|  |  | OR | 95% CI | OR | 95% CI | |
| Dietary costs | T1 (1,14-4,56€) | ref |  | ref |  | |
|  | T2 (4,57-5,58€) | 3.34** | 2.70; 4.14 | 3.26** | 2.61; 4.08 | |
|  | T3 (5,59-17,15€) | 3.82** | 2.96; 4.93 | 3.69** | 2.781; 4.84 | |
|  |  |  |  |  |  | |
| *Note.* We excluded the alcohol component from the total DHD15 index score (range: 0 – 120). High dietary quality was defined as the top quintile. *P<0.05; **P<0.001. DHD15 index \| Dutch Healthy Diet index 2015. T \| Tertile. Ref \| reference group. CI \| Confidence Interval. OR \| Odds ratio. | | | | | |  |
| ^a^ Model 1 is only adjusted for dietary energy \| ^b^ Model 2 additionally adjusts for age, sex, education, ethnicity, smoking and physical activity. | | | | | |  |

**Table S2:** association between daily dietary costs and dietary quality according to the MDS and DASH scores (n=4717).

| **Dietary quality (MDS – continuous score)** | | Model 1 ^a^ |  | Model 2 ^b^ |  | |
| --- | --- | --- | --- | --- | --- | --- |
|  |  | β | 95% CI | β | 95% CI | |
| Dietary costs | T1 (1,14-4,56€) | ref |  | ref |  | |
|  | T2 (4,57-5,58€) | 3.79** | 3.39; 4.18 | 2.97** | 2.59; 3.35 | |
|  | T3 (5,59-17,15€) | 4.74** | 4.26; 5.23 | 3.91** | 3.44; 4.38 | |
| **High dietary quality (MDS – dichotomous)** | | Model 1 ^a^ |  | Model 2 ^b^ |  | |
|  |  | OR | 95% CI | OR | 95% CI | |
| Dietary costs | T1 (1,14-4,56€) | ref |  | ref |  | |
|  | T2 (4,57-5,58€) | 6.48** | 4.81; 8.71 | 4.96** | 3.66; 6.71 | |
|  | T3 (5,59-17,15€) | 11.69** | 8.43; 16.21 | 9.18** | 6.54; 12.87 | |
|  |  |  |  |  |  | |
| **Dietary quality (DASH – continuous score)** | | Model 1 ^a^ |  | Model 2 ^b^ |  | |
|  |  | β | 95% CI | β | 95% CI | |
| Dietary costs | T1 (1,14-4,56€) | ref |  | ref |  | |
|  | T2 (4,57-5,58€) | 2.07** | 1.77; 2.38 | 1.79** | 1.47; 2.10 | |
|  | T3 (5,59-17,15€) | 2.70** | 2.40; 3.01 | 2.37** | 1.97; 2.76 | |
| **High dietary quality (DASH – dichotomous)** | | Model 1 ^a^ |  | Model 2 ^b^ |  | |
|  |  | OR | 95% CI | OR | 95% CI | |
| Dietary costs | T1 (1,14-4,56€) | ref |  | ref |  | |
|  | T2 (4,57-5,58€) | 2.22** | 1.89; 2.56 | 2.46** | 1.92; 3.15 | |
|  | T3 (5,59-17,15€) | 3.01** | 2.60; 3.42 | 2.88** | 2.16; 3.85 | |
| *Note.* High dietary quality was defined as the top quintile. MDS ranges from 0 to 80. DASH ranges from 7 to 35. *P<0.05; **P<0.001. DASH \| Dietary Approaches to Stop Hypertension. MDS \| Mediterranean Diet Score. T \| Tertile. Ref \| reference group. CI \| Confidence Interval. OR \| Odds ratio. | | | | | |  |
| ^a^ Model 1 is adjusted model for energy intake \|  ^b^ Model 2 further adjusts for age, sex, highest educational attainment, ethnicity, smoking status, and physical activity MET-minutes. | | | | | |  |

**Table S3:** Association between daily dietary costs in tertiles and (high) dietary quality excluding the alcohol component, by level of education (n=4717).

| **Dietary quality (DHD15 index – continuous score)** | | Lowest educated (n=1682) | | Medium educated (n=1343) | | Highest educated (n=1692) | |
| --- | --- | --- | --- | --- | --- | --- | --- |
|  |  | β | 95% CI | β | 95% CI | β | 95% CI |
| Dietary costs | T1 (1,14-4,56€) | ref |  | ref |  | ref |  |
|  | T2 (4,57-5,58€) | 7.54** | 5.68; 9.40 | 8.00** | 5.89; 10.12 | 7.97** | 6.05; 9.88 |
|  | T3 (5,59-17,15€) | 6.80** | 4.52; 9.07 | 8.91** | 6.24; 11.59 | 10.11** | 7.74; 12.47 |
| **High dietary quality (DHD15 index – dichotomous)** | | OR | 95%CI | OR | 95%CI | OR | 95%CI |
| Dietary costs | T1 (1,14-4,56€) | ref |  | ref |  | ref |  |
|  | T2 (4,57-5,58€) | 3.71** | 2.55; 5.39 | 2.63** | 1.71; 4.03 | 3.63** | 2.48; 5.30 |
|  | T3 (5,59-17,15€) | 3.56** | 2.43; 5.66 | 3.27* | 1.92; 5.55 | 4.42** | 2.83; 6.92 |
| *Note.* We excluded the alcohol component from the total DHD15 index score (range: 0 – 120). High dietary quality was defined as the top quintile. *P<0.05; **P<0.001. DHD15 index \| Dutch Healthy Diet index 2015. T \| Tertile. Ref \| reference group. CI \| Confidence Interval. | | | | | | | |
| All models adjust for age, sex, ethnicity, smoking, energy intake and physical activity. | | | | | | | |

**Table S4:** Association between daily dietary costs and dietary quality according to the MDS and DASH scores by level of education (n=4717).

| **Dietary quality (MDS – continuous score)** | | Low educated  (n=1682) | | Medium educated (n=1343) | | High educated (n=1692) | |
| --- | --- | --- | --- | --- | --- | --- | --- |
|  |  | β | 95% CI | β | 95% CI | β | 95% CI |
| Dietary costs | T1 (1,14-4,56€) | ref |  | ref |  | ref |  |
|  | T2 (4,57-5,58€) | 2.98** | 2.33; 3.64 | 3.06** | 2.36; 3.76 | 2.85** | 2.23; 3.47 |
|  | T3 (5,59-17,15€) | 3.86** | 3.06; 4.67 | 4.37** | 3.49; 5.26 | 3.64** | 2.87; 4.40 |
| **Dietary quality (DASH – continuous score)** | | Low educated  (n=1682) | | Medium educated (n=1343) | | High educated (n=1692) | |
|  |  | β | 95% CI | β | 95% CI | β | 95% CI |
| Dietary costs | T1 (1,14-4,56€) | ref |  | ref |  | ref |  |
|  | T2 (4,57-5,58€) | 1.73** | 1.19; 2.27 | 1.97** | 1.37; 2.57 | 1.68** | 1.16; 2.21 |
|  | T3 (5,59-17,15€) | 1.96** | 1.30; 2.61 | 3.14** | 2.39; 3.90 | 2.17** | 1.52; 2.82 |
| *Note.* MDS ranges from 0 to 80. DASH ranges from 7 to 35. *P<0.05; **P<0.001. DASH \| Dietary Approaches to Stop Hypertension. MDS \| Mediterranean Diet Score. T \| Tertile. Ref \| reference group. CI \| Confidence Interval. | | | | | | | |
| All models adjust for age, sex, ethnicity, smoking status, energy intake and physical activity MET-minutes. | | | | | | | |

**Table S5:** Association between daily dietary costs in tertiles and dietary quality excluding the alcohol component, by ethnicity (n=4717).

| **Dietary quality (DHD15 index – continuous score)** | | Dutch  (n=1429) | | South-Asian Surinamese  (n=1003) | | African Surinamese  (n=980) | | Moroccan  (n=717) | | Turkish  (n=588) | |
| --- | --- | --- | --- | --- | --- | --- | --- | --- | --- | --- | --- |
|  |  | β | 95% CI | Β | 95% CI | β | 95% CI | β | 95% CI | β | 95% CI |
| Dietary costs | T1 (1,14-4,56€) | ref |  | ref |  | ref |  | ref | | ref |  |
|  | T2 (4,57-5,58€) | 6.69** | 4.49; 8.89 | 7.78** | 5.43; 10.13 | 8.20** | 5.83; 10.56 | 6.25** | 3.38; 9.12 | 10.04** | 6.78; 13.29 |
|  | T3 (5,59-17,15€) | 9.12** | 6.40; 11.84 | 9.07** | 5.98; 12.16 | 9.52** | 6.62; 12.42 | 5.04* | 1.37; 8.71 | 8.35** | 4.69; 12.01 |
| **High dietary quality (DHD15 index – dichotomous)** | | OR | 95% CI | OR | 95% CI | OR | 95% CI | OR | 95% CI | OR | 95% CI |
| Dietary costs | T1 (1,14-4,56€) | Ref |  | Ref |  | ref |  | ref |  | ref |  |
|  | T2 (4,57-5,58€) | 2.99** | 1.90; 4.70 | 2.59** | 1.65; 4.06 | 3.85** | 2.22; 6.68 | 3.15** | 1.93; 5.16 | 7.78** | 3.44; 17.63 |
|  | T3 (5,59-17,15€) | 3.93** | 2.31; 6.67 | 3.45** | 1.93’6.16 | 5.29** | 2.77; 10.11 | 1.87 | 0.97; 3.62 | 6.78** | 2.76; 16.68 |
| *Note.* We excluded the alcohol component from the total DHD15 index score (range: 0 – 120). High dietary quality was defined as the top quintile. *P<0.05; **P<0.001. DHD15 index \| Dutch Healthy Diet index 2015. T \| Tertile. Ref \| reference group. CI \| Confidence Interval. | | | | | | | | | | | |
| All models adjust for age, sex, education, smoking, energy intake and physical activity. | | | | | | | | | | | |

**Table S6:** association between dietary costs and dietary quality according to the MDS and DASH scores by ethnicity (n=4717).

| **Dietary quality (MDS – continuous score)** | | Dutch (n=1429) | | South Asian Surinamese (n=1003) | | African Surinamese (n=980) | | Moroccan (n=717) | | Turkish (n=588) | |
| --- | --- | --- | --- | --- | --- | --- | --- | --- | --- | --- | --- |
|  |  | β | 95% CI | β | 95% CI | β | 95% CI |  |  |  |  |
| Dietary costs | T1 (1,14-4,56€) | ref |  | ref |  | ref |  | ref | | ref |  |
|  | T2 (4,57-5,58€) | 2.77** | 2.10; 3.43 | 3.70** | 2.87; 4.53 | 2.66** | 1.80; 3.52 | 2.04** | 1.14; 2.93 | 3.80** | 2.59; 5.02 |
|  | T3 (5,59-17,15€) | 3.77** | 2.95; 4.59 | 5.48** | 4.39; 6.57 | 4.04** | 2.98; 5.09 | 2.35** | 1.21; 3.49 | 3.77** | 2.41; 5.14 |
| **Dietary quality (DASH – continuous score)** | | Dutch (n=1429) | | South Asian Surinamese (n=1003) | | African Surinamese (n=980) | | Moroccan  (n=717) | | Turkish (n=588) | |
|  |  | β | 95% CI | β | 95% CI | β | 95% CI | β | 95% CI | β | 95% CI |
| Dietary costs | T1 (1,14-4,56€) | ref |  | ref |  | ref |  | ref |  | Ref |  |
|  | T2 (4,57-5,58€) | 1.65** | 1.08; 2.23 | 1.63** | 0.93; 2.32 | 2.44** | 1.75; 3.11 | 1.22* | 0.43; 2.01 | 1.88** | 0.89; 2.87 |
|  | T3 (5,59-17,15€) | 2.40** | 1.68; 3.12 | 2.23** | 1.31; 3.15 | 3.13** | 2.30; 3.97 | 1.42* | 0.41; 2.42 | 1.92* | 0.80; 3.03 |
| *Note.* MDS ranges from 0 to 80. DASH ranges from 7 to 35. *P<0.05; **P<0.001. DASH \| Dietary Approaches to Stop Hypertension. MDS \| Mediterranean Diet Score. T \| Tertile. Ref \| reference group. CI \| Confidence Interval. | | | | | | | | | | | |
| All models adjust for age, sex, ethnicity, smoking status, energy intake and physical activity MET-minutes. | | | | | | | | | | | |
